# Total-Body Dynamic Imaging and Kinetic Modeling of [^18^F]F-AraG in Healthy Individuals and a Non–Small Cell Lung Cancer Patient Undergoing Anti–PD-1 Immunotherapy

**DOI:** 10.2967/jnumed.123.267003

**Published:** 2024-09

**Authors:** Negar Omidvari, Jelena Levi, Yasser G. Abdelhafez, Yiran Wang, Lorenzo Nardo, Megan E. Daly, Guobao Wang, Simon R. Cherry

**Affiliations:** 1 Department of Biomedical Engineering, University of California Davis, Davis, California;; 2 CellSight Technologies Inc., San Francisco, California;; 3 Department of Radiology, University of California Davis Medical Center, Sacramento, California;; 4 Radiotherapy and Nuclear Medicine Department, South Egypt Cancer Institute, Assiut University, Asyut, Egypt; and; 5 Department of Radiation Oncology, University of California Davis Comprehensive Cancer Center School of Medicine, Sacramento, California

**Keywords:** T cells, immunotherapy, NSCLC, kinetic modeling, total-body PET

## Abstract

Immunotherapies, especially checkpoint inhibitors such as anti–programmed cell death protein 1 (anti–PD-1) antibodies, have transformed cancer treatment by enhancing the immune system’s capability to target and kill cancer cells. However, predicting immunotherapy response remains challenging. ^18^F-arabinosyl guanine ([^18^F]F-AraG) is a molecular imaging tracer targeting activated T cells, which may facilitate therapy response assessment by noninvasive quantification of immune cell activity within the tumor microenvironment and elsewhere in the body. The aim of this study was to obtain preliminary data on total-body pharmacokinetics of [^18^F]F-AraG as a potential quantitative biomarker for immune response evaluation. **Methods:** The study consisted of 90-min total-body dynamic scans of 4 healthy subjects and 1 non–small cell lung cancer patient who was scanned before and after anti–PD-1 immunotherapy. Compartmental modeling with Akaike information criterion model selection was used to analyze tracer kinetics in various organs. Additionally, 7 subregions of the primary lung tumor and 4 mediastinal lymph nodes were analyzed. Practical identifiability analysis was performed to assess the reliability of kinetic parameter estimation. Correlations of the SUV_mean_, the tissue-to-blood SUV ratio (SUVR), and the Logan plot slope (*K*_Logan_) with the total volume of distribution (*V*_T_) were calculated to identify potential surrogates for kinetic modeling. **Results:** Strong correlations were observed between *K*_Logan_ and SUVR with *V*_T_, suggesting that they can be used as promising surrogates for *V*_T_, especially in organs with a low blood-volume fraction. Moreover, practical identifiability analysis suggested that dynamic [^18^F]F-AraG PET scans could potentially be shortened to 60 min, while maintaining quantification accuracy for all organs of interest. The study suggests that although [^18^F]F-AraG SUV images can provide insights on immune cell distribution, kinetic modeling or graphical analysis methods may be required for accurate quantification of immune response after therapy. Although SUV_mean_ showed variable changes in different subregions of the tumor after therapy, the SUVR, *K*_Logan_, and *V*_T_ showed consistent increasing trends in all analyzed subregions of the tumor with high practical identifiability. **Conclusion:** Our findings highlight the promise of [^18^F]F-AraG dynamic imaging as a noninvasive biomarker for quantifying the immune response to immunotherapy in cancer patients. Promising total-body kinetic modeling results also suggest potentially wider applications of the tracer in investigating the role of T cells in the immunopathogenesis of diseases.

## Visual Abstract

**Figure:**
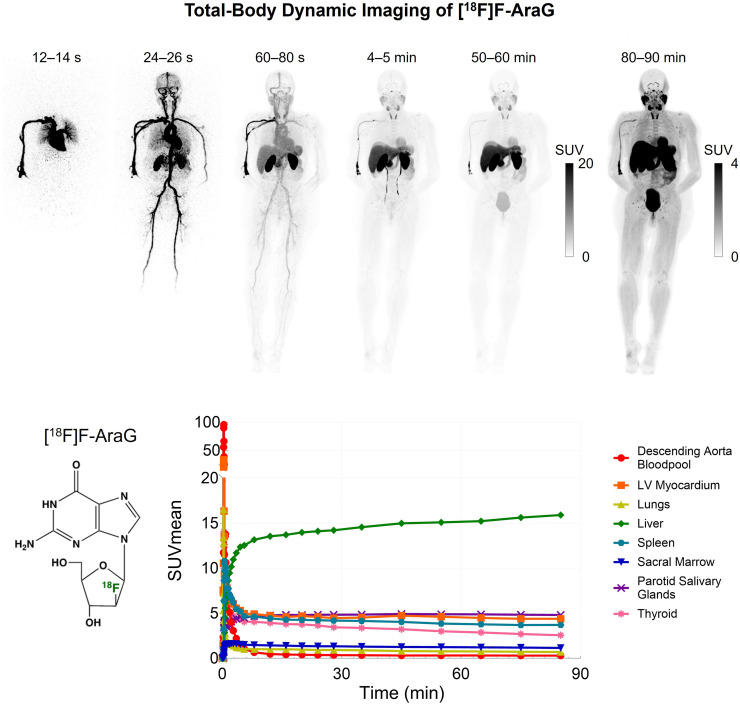

Immunotherapy has revolutionized cancer treatment by enhancing the capability of the patient’s immune system to target and eliminate cancer cells. One of the most widely used immunotherapy classes is immune checkpoint inhibitors targeting the programmed cell death protein 1 (PD-1) or programmed death ligand 1 (PD-L1) axis. These antibodies disrupt the inhibitory signals between cancer cells and T cells to reinvigorate the immune response. Despite the remarkable clinical successes of anti–PD-1 and anti–programmed death ligand 1 immunotherapies, not all patients respond equally to treatment, highlighting the need for early predictive biomarkers to identify potential responders. Predicting immunotherapy response is challenging because of the complexity of the tumor microenvironment and the systemic nature of the immune response, particularly involving the lymph nodes (LNs). The dynamic nature of the immune response necessitates a comprehensive understanding of the spatiotemporal distribution of immune cells within the body, especially in tumors and tumor-draining LNs (1*,*^2^).

In recent years, molecular imaging techniques, such as PET, have emerged as powerful tools for enhanced understanding of the complex interactions between the immune system and cancer. Specifically, development of novel radiotracers targeting specific immune cells has introduced new means for noninvasive quantification of immune cell distribution within the tumor microenvironment (3*,*^4^). Among these radiotracers, ^18^F-arabinosyl guanine ([^18^F]F-AraG) has garnered significant attention because of its selectivity toward activated T cells and its small molecular size.

[^18^F]F-AraG is the ^18^F-labeled analog of AraG, developed as a PET tracer for imaging T-cell activation (5). The prodrug of AraG, nelarabine, is approved by the U.S. Food and Drug Administration for treatment of T-cell acute lymphoblastic leukemia and T-cell lymphoblastic lymphoma and has been extensively studied for its selective T-cell toxicity (6). Similarly to AraG, [^18^F]F-AraG enters the cells via nucleoside transporters and is phosphorylated by 2 enzymes: primarily by mitochondrial deoxyguanosine kinase (dGK), a critical enzyme for mitochondrial DNA synthesis, and to a lesser extent by cytoplasmic deoxycytidine kinase. Triphosphorylated [^18^F]F-AraG can be incorporated into mitochondrial DNA or can exit mitochondria and become dephosphorylated by the sterile α motif and histidine-aspartate domain–containing protein 1 (SAMHD1), allowing efflux of [^18^F]F-AraG. Cells requiring increased DNA synthesis, such as activated T cells, exhibit downregulation of SAMHD1. The increased mitochondrial mass and mitochondrial DNA synthesis and downregulation of SAMHD1 in activated T cells result in significantly increased uptake and retention of [^18^F]F-AraG by activated T cells compared with their unstimulated state, enabling the use of [^18^F]F-AraG as an in vivo probe to assess T-cell activation (7*,*^8^).

[^18^F]F-AraG has considerable uptake in almost all organs, depending on expression and activity profiles of dGK and SAMHD1 in different cell types present in the organ (8). However, in the context of antitumor immune response evaluation, cancer cells other than malignancies of the T-cell lineage are expected to have low uptake and retention of [^18^F]F-AraG, suggesting a correlation of [^18^F]F-AraG tumor uptake with the abundance and activation state of immune cells in the tumor microenvironment. This is partly linked to the expression of SAMHD1 in cancer cells. Among a wide range of investigated cancer cell lines in the Cancer Cell Line Encyclopedia, T-cell acute lymphoblastic leukemia cells show significantly lower expression of SAMHD1, which has been linked to AraG’s selectivity for activated T cells (9). Among human immune cells, the highest [^18^F]F-AraG uptake has been observed in T cells, macrophages, and dendritic cells, with T cells showing the highest retention in addition to significantly higher uptake on activation (7). Consistent with these findings, previous preclinical studies of [^18^F]F-AraG in a range of tumor models in mice have shown correlations of [^18^F]F-AraG tracer uptake with the frequency of CD8^+^ PD-1^+^ T cells in the tumors (10).

Previous studies using [^18^F]F-AraG have demonstrated its potential in providing insights into the spatial distribution of tumor-infiltrating immune cells (7). However, the dynamic behavior of [^18^F]F-AraG within the body and its kinetic parameters remain unexplored. This study is the first report, to our knowledge, on dynamic imaging and kinetic modeling of [^18^F]F-AraG, as well as being the first-in-human report on using [^18^F]F-AraG in a non–small cell lung cancer (NSCLC) patient undergoing anti–PD-1 immunotherapy, aiming to enhance our understanding of its potential quantitative utility in predicting immunotherapy response, as well as its wider application in other disease conditions.

## MATERIALS AND METHODS

### Study Design

The study consisted of 5 subjects, including 4 healthy controls and 1 NSCLC patient. The demographics of the subjects and their medical history are provided in Supplemental Table 1 (supplemental materials are available at http://jnm.snmjournals.org). The protocol was approved by the University of California Davis Institutional Review Board (#1630355), and all participants provided written informed consent.

The NSCLC patient had metastatic stage IV NSCLC adenocarcinoma of the left lower lobe (LLL) and right lower lobe (RLL), both diagnosed as primary tumors, histologically confirmed through bronchoscopy (PD-L1 < 1%). Both lesions were larger than 1 cm in size, and the LLL mass was sufficiently separated from other organs with known high [^18^F]F-AraG uptake, so that quantification was feasible. Quantification in the RLL was not feasible because of the proximity of the lesion to the liver. Fine-needle aspiration biopsy from 1 of 3 mediastinal LNs suggested metastatic carcinoma in station 4L. The patient was enrolled in a clinical trial and received pembrolizumab anti–PD-1 immunotherapy combined with pemetrexed–carboplatin chemotherapy every 3 wk. Before the third immunotherapy cycle, the patient received a course of stereotactic body radiation therapy to the LLL lung with palliative intent. More details on the study design are provided in the supplemental materials. The oncologic history of the NSCLC patient and the timeline of the medical interventions of the patient with respect to [^18^F]F-AraG scans are available in an online repository at Harvard Dataverse (11).

### Radiotracer Administration and PET/CT Imaging

[^18^F]F-AraG was synthesized by Optimal Tracers. Participants received an intravenous bolus injection (<2-s duration) of approximately 189 MBq (mean, 189.0 ± 12.6 MBq; range, 168.1–200.6 MBq) of [^18^F]F-AraG. The expected total effective radiation dose was 0.0167 mSv/MBq for women and 0.0157 mSv/MBq for men, based on the previously reported dosimetry study (8). Subjects had 90-min total-body dynamic PET scans with [^18^F]F-AraG on the uEXPLORER scanner (United Imaging Healthcare) starting immediately before the intravenous bolus injection, except for subject 3, whose scan was terminated after 60 min because of patient discomfort and motion. A low-dose CT scan (dose-modulated, maximum 50 average mAs, 140 kVp, 10-mSv estimated effective radiation dose) was performed before the PET scan of each subject for attenuation correction and anatomic correlation. The cancer patient was first scanned 1 d before the first immunotherapy cycle and again 14 d later after receiving the first cycle. The image reconstruction protocol and the kernel-based postreconstruction smoothing (12) used on PET datasets are described in the supplemental materials.

The initial staging of the cancer patient included a PET/CT scan performed 110 d before immunotherapy on a Discovery 690 scanner (GE Healthcare) 56 min after a 375.7-MBq intravenous bolus injection of [^18^F]FDG. Additionally, 2 sets of contrast-enhanced chest CT scans, with intravenous administration of 100 mL of Omnipaque 350 (GE Healthcare), performed on a Revolution EVO scanner (GE Healthcare), 2 d before therapy and 82 d after therapy (after 5 immunotherapy cycles), were used for initial therapy response evaluation.

### Image Analysis

An in-house–developed code package in MATLAB R2021b (MathWorks) (described in the supplemental materials) was used to generate segmented regions for the blood pool (descending aorta and right ventricle [RV]), cerebrum, choroid plexus, pituitary gland, salivary glands, thyroid, bone marrow, lungs, myocardium (RV and left ventricle [LV]), spleen, liver, kidneys, femoral muscle, and LNs (axillary and pelvic). For individual subjects, additional spheric volumes of interest (VOIs) were placed in regions with high focal uptake. For the NSCLC patient, 7 spheric VOIs were placed on different subregions of the patient’s LLL tumor to cover the tumor volume while including a variety of low-uptake and high-uptake subregions within the tumor. Moreover, 4 spheric VOIs were placed on the patient’s high-uptake mediastinal LNs, 2 of which were identified as enlarged, likely metastatic, LNs. The SUV was expressed as SUV_mean_ in all organs, calculated from the average of the voxels within each segmented organ, except for LNs, for which SUV_peak_ was used because of their small size. In the tumor subregions, SUV_mean_ was calculated directly from individual spheric VOIs placed on each subregion. SUV_peak_ was defined as the mean of the 8 hottest voxels within the VOI, equivalent to a volume of 100 mm^3^.

### Kinetic Modeling

The descending aorta blood pool was used as the input function for all organs, except for the lungs and the lung tumor subregions, for which the RV blood pool was used to represent the blood-pool activity concentration in the pulmonary artery as the dominant blood supply of the lungs. Three compartmental models were fitted on each time–activity curve to select the model best describing the kinetics in each organ. This included the 1-tissue compartmental model with 4 fitting microparameters (1T4P) (*v*_b_, *K*_1_, *k*_2_, and *t*_d_), the 2-tissue compartmental model with 5 fitting microparameters (2T5P) (*v*_b_, *K*_1_, *k*_2_, *k*_3_, and *t*_d_), and the 2-tissue compartmental model with 6 fitting microparameters (2T6P) (*v*_b_, *K*_1_, *k*_2_, *k*_3_, *k*_4_, and *t*_d_), in which *v*_b_ is the fractional blood volume; *K*_1_, *k*_2_, *k*_3_, and *k*_4_ are the rate constants between model compartments; and *t*_d_ is the time delay. The Akaike information criterion (AIC) with a correction for small sample sizes was used to choose the model best fitting the data (13*,*^14^). The estimated time delays were used to delay-correct the blood-pool time–activity curves for each organ, and the data points from 14 to 90 min were fitted with a biexponential function and extrapolated to 10 h after injection to model the tissue time–activity curves at equilibrium time.

Tissue-to-blood SUV ratio (SUVR) was calculated from the ratio of the tissue activity concentration in each organ (*C*_T_) to the whole-blood time–activity concentration (*C*_P_). Net influx rate, $K_{i}\;=\frac{K_{1}k_{3}}{k_{2}+k_{3}}$, was calculated for the 2-tissue model. Total volume of distribution, *V*_T_, was calculated as $\frac{K_{1}}{k_{2}}(1+\frac{k_{3}}{k_{4}})$ and $\frac{K_{1}}{k_{2}}$ for the 2T6P and the 1T4P models, respectively (15). Since in the 2T6P model, SUVR at equilibrium time should reach the blood-volume–corrected *V*_T_, defined as ${V_{T(v_{b})}=\;v}_{b}+V_{T}\left(1-v_{b}\right)$, SUVR was estimated up to 10 h after injection, using the extrapolated blood-pool time–activity curves, and compared with $V_{T\left(v_{b}\right)}$ to evaluate the equilibrium time in different organs. Lastly, Logan plot slopes (*K*_Logan_) (the linear regression between $\int_{\tau =0}^{t}C_{T}d\tau /C_{T}$ and $\int_{\tau =0}^{t}C_{P}d\tau /C_{T}$) were generated, and *K*_Logan_ was calculated from the slope of linear fits on data points after 30 min (t* = 30 min) (16).

To determine whether model parameters can be accurately estimated in the presence of noise, practical identifiability analysis was performed, including calculation of normalized sensitivity curves, bias, SD, and root mean square error (RMSE) of the model parameters using 100 simulated noisy time–activity curves (17). The practical identifiability analysis was repeated using the data points from the first 60 min only, and bias, SD, and RMSE of the model parameters were calculated in reference to the parameter estimations from the 90-min data fitting to investigate kinetic modeling feasibility in future studies using 60-min dynamic scans. A detailed description of the kinetic modeling methods is provided in the supplemental materials.

### Statistical Analysis

Spearman rank correlation coefficients were calculated in MATLAB R2021b between the *V*_T_ and the 3 parameters SUV, SUVR, and *K*_Logan_ to investigate their use as surrogates of *V*_T_. Additionally, correlation coefficients of $V_{T\left(v_{b}\right)}$ with SUVR and *K*_Logan_ were calculated for model validation and to investigate the effects from variabilities in tissue fractional blood volume. Hypothesis testing, comparing the pretherapy and posttherapy scans of the NSCLC patient, was performed for the LLL tumor and the LNs in GraphPad Prism version 10.0, using a Wilcoxon paired signed-rank test. *P* values of less than 0.05 were set to determine the statistical significance. No multiple-comparison correction was included.

## RESULTS

### Biodistribution in Healthy Tissues

**FIGURE 1.**
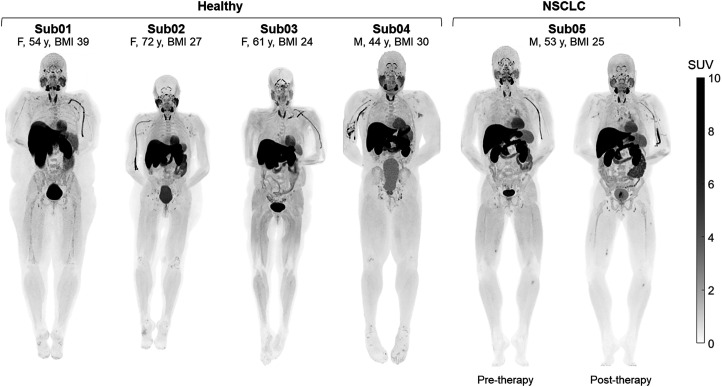
Maximum-intensity projections of PET SUV images (50–60 min after injection) of 4 healthy subjects and 1 NSCLC patient scanned before and after therapy. BMI = body mass index; Sub = subject.

**FIGURE 2.**
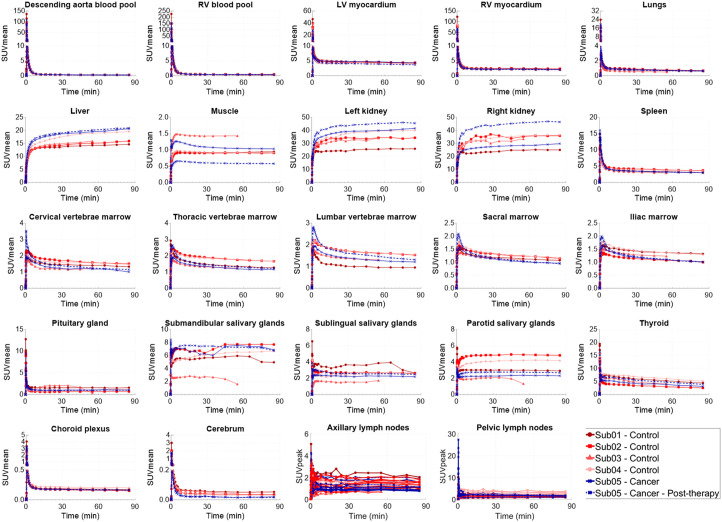
Time–activity curves of healthy organs of interest analyzed in all subjects, showing complete duration of 90-min dynamic scans. Sub = subject.

Static SUV images (Fig. 1) showed the highest uptake in the liver, kidneys, and urinary bladder, followed by the stomach wall, pancreas, salivary glands, thyroid, myocardium, spleen, bone marrow, choroid plexus, pituitary gland, ocular muscles, adrenal gland, and LNs. Prominent uptake was observed in pelvic and axillary LNs of all subjects. Uptake in the cerebrum and cerebellum was negligible in all subjects, with an SUV_mean_ below 0.06 and 0.08 for times more than 30 min after injection, respectively. Time–activity curves of the healthy organs of interest showed consistent trends (90-min time–activity curves are shown in Fig. 2, and zoomed-in versions of the first 2 min for all analyzed regions of interest are shown in Supplemental Fig. 1), with a decrease in SUV_mean_ for times approaching 90 min, except for the liver and kidneys, in which radiotracer excretion effects are expected. Motion artifacts observed on time–activity curves of 2 subjects toward the scan end particularly affected the quantification in the salivary glands of subjects 1 and 3 and the pituitary gland in subject 3. Therefore, regions affected by motion were excluded from further analysis. Time–activity curves of the descending aorta blood pool were compared in all participants using both SUV_mean_ and the percentage of injected dose per liter, and biologic half-lives derived from biexponential fitting on the whole-blood time–activity curves are shown in Supplemental Figure 2. Subjects showed variable levels of activity in their bowel; however, in most cases, it was not possible to confirm whether the activity was in the lumen or the bowel wall, except for subject 3, in whom cross-sectional analysis suggested uptake in sections of the ascending colon wall (Supplemental Fig. 3A).

### Incidental Findings

**FIGURE 3.**
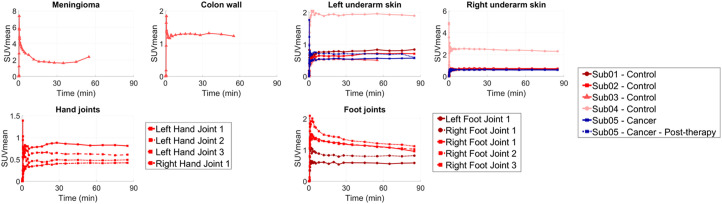
Time–activity curves of organs with focally high uptake pattern in all subjects, showing complete duration of 90-min dynamic scans. Meningioma time–activity curve was affected by head motion for times later than 30 min after injection. Sub = subject.

Control subjects showed increased focal uptake with altered kinetics at sites consistent with a range of preexisting clinical conditions in their medical records. The bilateral underarm skin region showed increased uptake and skin thickening compared with other skin regions in all subjects, and the underarm skin uptake in subject 4, who had an asymptomatic chronic viral infection, was higher than that of the other subjects by a factor of 2. Subject 1, who had a history of pain in the arch of bilateral feet, showed corresponding focal uptake in bilateral foot joints. Subject 2, with history of osteoarthritis, showed focal uptakes in the joints of the hands and the feet. Subject 3, with a middle cranial fossa complex meningioma, showed correspondingly high uptake in the meningioma. The time–activity curves of the tissues that showed increased uptake compared with their healthy state showed distinct kinetics, with plateauing or increasing uptake 60 min after injection (Fig. 3). PET/CT images of the incidental findings are shown in Supplemental Figures 3–4.

### Uptake in NSCLC Tumor and Mediastinal LNs

**FIGURE 4.**
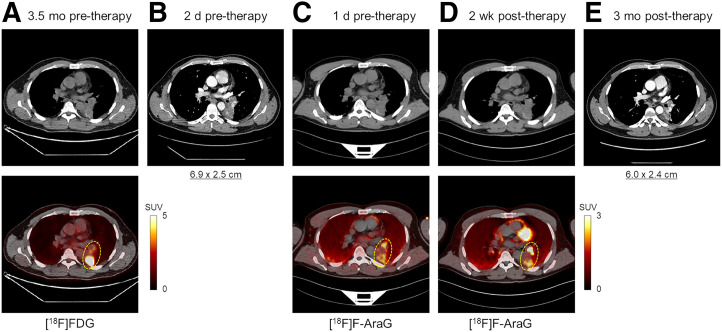
Transverse slices through LLL mass of subject 5, comparing initial [^18^F]FDG PET/CT 3.5 mo before therapy (A), contrast-enhanced CT 2 d before therapy (B), [^18^F]F-AraG PET/CT 1 d before therapy (C), [^18^F]F-AraG PET/CT 2 wk after therapy (D), and contrast-enhanced CT 3 mo after therapy (E).

**FIGURE 5.**
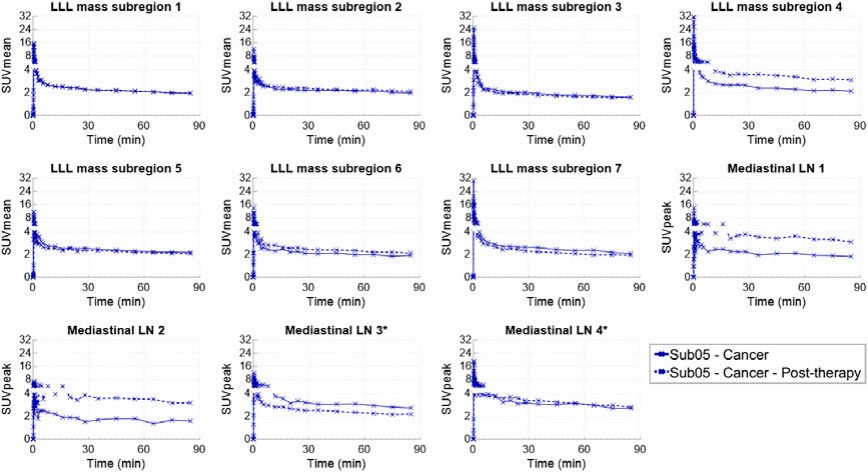
Time–activity curves of 7 subregions of LLL mass and 4 mediastinal LNs, showing complete duration of 90-min dynamic scans. *Two enlarged LNs. Sub = subject.

The LLL mass of the NSCLC patient showed a slight size reduction after therapy, from 6.9 × 2.5 cm to 6.0 × 2.4 cm measured on the contrast-enhanced CTs (Fig. 4). The 2 enlarged, presumably metastatic, mediastinal LNs also showed slight size reduction after therapy from 2.1 × 1.4 cm to 1.5 × 0.9 cm and from 2.1 × 1.5 cm to 1.7 × 1.2 cm. The [^18^F]FDG pretherapy scan and the [^18^F]F-AraG scans showed heterogeneous uptake in the LLL mass; however, the uptake patterns differed between the 2 tracers. The [^18^F]FDG scan showed substantially higher uptake in the posterior section of the tumor (SUV_max_, 14.8) than in its other subregions (SUV_max_, 4.0), whereas the 2 [^18^F]F-AraG scans showed high-uptake subregions in both the anterior and the posterior subregions of the tumor with similar intensities, and some subregions of the tumors with moderate [^18^F]FDG uptake showed no quantifiable uptake on the [^18^F]F-AraG scans (Fig. 4). Furthermore, the 2 nonenlarged high-uptake mediastinal LNs on the [^18^F]F-AraG scans did not show quantifiable uptake on the [^18^F]FDG scan. The 7 subregions of the LLL tumor selected for analysis and the 4 mediastinal LNs, 2 of which were identified as enlarged LNs, showed similar trends of kinetics with a decreasing uptake between 30 and 90 min (Fig. 5). PET/CT images of the analyzed subregions of the tumor and the selected mediastinal LNs are shown in Supplemental Figures 5–9. Comparison of the SUV_mean_ of the 7 tumor subregions showed no trend in SUV_mean_ changes after therapy, varying from −12% to +45%. Similarly, the 4 mediastinal LNs showed a wide range of SUV_peak_ changes after therapy, varying from −22% to +112%.

### Compartmental Modeling of the [^18^F]F-AraG Kinetics

Single–input-function kinetic modeling with 1-tissue and 2-tissue compartmental models with time-delay estimation successfully fitted the time–activity curves in all analyzed organs of interest, except for the liver and RV myocardium, in which the initial time–activity curve peaks suffered from poor fitting. AIC favored the 2T6P model in all analyzed tumor subregions, mediastinal LNs, and the analyzed healthy organs of interest, except for axillary and pelvic LNs and RV myocardium. In 70 of the 93 total analyzed axillary and pelvic LNs, the AIC favored the 2T6P model, whereas the 2T5P and the 1T4P models were favored in 10 and 13 LNs, respectively. Additionally, in RV myocardium, AIC favored the 1T4P model in all subjects. The averaged parameter estimates calculated from model fitting performed on 90-min and 60-min dynamic data for all analyzed organs of interest are summarized in Supplemental Tables 2 and 3, respectively, and show negligible differences in parameter estimates from 60-min and 90-min dynamic data.

Normalized sensitivity plots showed that *K*_1_ and *v*_b_ reach their peak sensitivity during the first 1 min. Although the sensitivity of the time–activity curve to *k*_4_ showed a continuous increasing trend during the 90-min scans in all analyzed organs of interest, sensitivities to *k*_2_ and *k*_3_ varied in different organs. Practical identifiability analysis with joint time-delay estimation showed low biases (−5.3% to +4.2%) in *V*_T_ estimation in all analyzed organs of interest. SD and RMSE of *V*_T_ estimates were also low (2.4%–10.3%) in most organs of interest, except for the RV myocardium and muscle, in which SD and RMSE of *V*_T_ estimates increased up to 24.7% and 29.3%, respectively. The individual microparameter estimates showed higher variability compared with *V*_T_. Among all microparameters, *v*_b_ showed the highest bias and variability. *K*_1_ showed low biases (−6.5% to +8.5%) in most organs, except in the lungs and salivary glands, in which the *K*_1_ bias was 18.7% and −15.5%, respectively; however, SD and RMSE of *K*_1_ estimates were still higher than those with *V*_T_ and were particularly high in the lungs and choroid plexus. In a number of organs, all rate constants showed low biases (within ±10%). This included LV myocardium, RV myocardium, spleen, thyroid, bone marrow, underarm skin, LLL tumor subregions, and mediastinal LNs; however, SD and RMSE of the rate-constant estimates were low (within ±10%) in only the RV myocardium.

Repeating the practical identifiability analysis by setting the time delays to their preestimated values substantially improved the bias and variability of the model microparameters in all organs of interest. Furthermore, repeating the practical identifiability analysis with joint time-delay estimation and using the data points from only the first 60 min for fitting showed that bias, SD, and RMSE in all parameter estimates were similar to those using the whole 90-min datasets.

### SUVR and K_Logan_ as Surrogates for V_T_

SUVR curves showed a plateauing trend toward the end of the 90-min dynamic scans in most analyzed organs of interest, except for the liver and kidneys, which showed an increasing trend, and the thyroid, which showed a decreasing trend. The 60-min to 90-min SUVRs showed increases (+4% to +71%) in all 7 subregions of the LLL tumor after therapy. The 2 enlarged mediastinal LNs showed decreased SUVRs after therapy (−33% and −10%), whereas the 2 nonenlarged mediastinal LNs showed an increasing trend after therapy (48% and 82%). Comparing the 10-h extrapolated SUVR curves to the $V_{T(v_{b})}$ showed smaller differences between the SUVR curves and $V_{T(v_{b})}$ at 10 h than at 90 min after injection in all organs of interest (Supplemental Figs. 7 and 8). Furthermore, Logan plots showed a linear slope in all organs of interest 30 min after injection.

**FIGURE 6.**
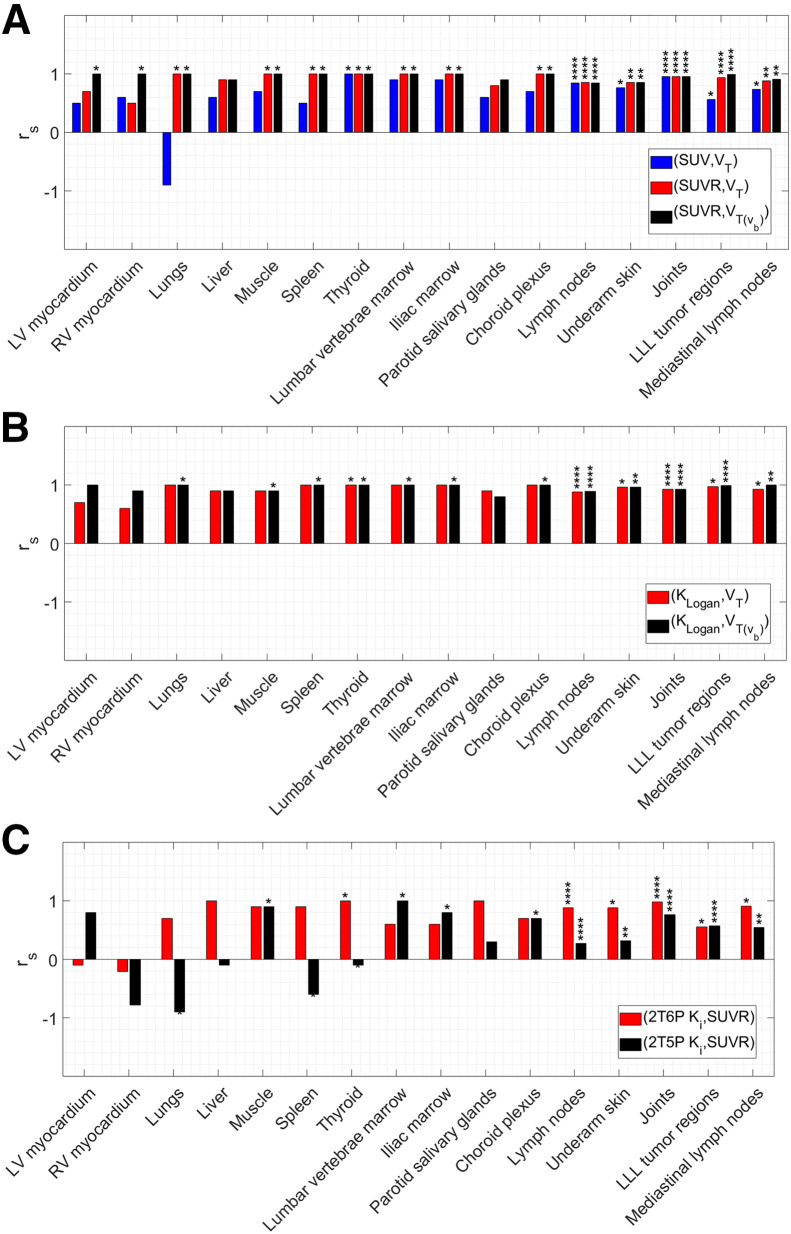
Spearman rank correlation coefficients (r_s_) calculated between (SUV, *V*_T_), (SUVR, *V*_T_), and (SUVR, $V_{T\left(v_{b}\right)}$) (A), between (*K*_Logan_, *V*_T_) and (*K*_Logan_,$\;V_{T\left(v_{b}\right)}$) (B), and between (2T6P *K_i_*, SUVR) and (2T5P *K_i_*, SUVR) (C) in different organs of interest. Asterisks shown above individual bars show significance level of Spearman rank *P* values (*P < 0.05, **P < 0.01, ***P < 0.001, ****P < 0.0001).

Both *K*_Logan_ and SUVR from 60 to 90 min after injection were highly correlated with $V_{T(v_{b})}$ in all organs of interest, with correlation coefficients within 0.80–1.00 and 0.85–1.00, respectively (Fig. 6). Similarly, *K*_Logan_ and SUVR were highly correlated with *V*_T_ in most organs of interest, showing slightly lower correlation coefficients (<5%) compared with $V_{T(v_{b})}$ correlations, except for LV and RV myocardium and parotid salivary glands, in which correlation coefficients with *V*_T_ were substantially lower than with $V_{T(v_{b})}$. SUVs from 60 to 90 min after injection also showed moderate to strong correlations with *V*_T_ in some organs, including the thyroid, bone marrow, LNs, underarm skin, and joints, with correlation coefficients within 0.74–1.00. However, in other organs, correlations were weak, and in the case of the lungs, the SUV_mean_ was negatively correlated with *V*_T_. Correlations of *K_i_* obtained from the 2T6P and 2T5P models with SUVR also showed inconsistent results, ranging from negative correlations in some organs and tissues (e.g., myocardium, lungs, and thyroid) to moderate or strong positive correlations in others (e.g., joints, LNs, and tumor subregions).

**FIGURE 7.**
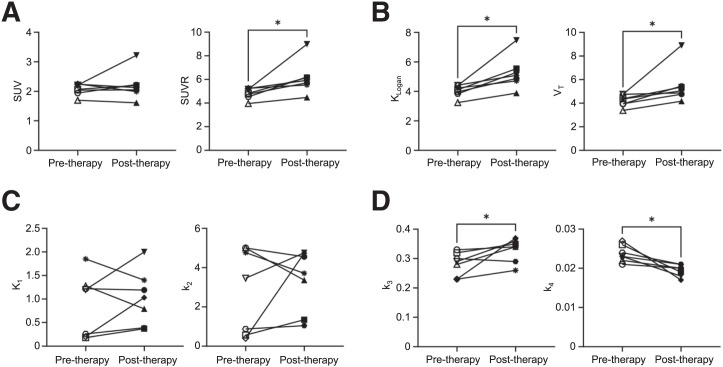
Changes in 7 subregions of LLL tumor after therapy, using SUV_mean_ or SUVR without kinetic modeling (A), *K*_Logan_ from Logan plots or *V*_T_ from 2T6P compartmental modeling as macroparameters with high identifiability (B), 2T6P rate constants between blood and first compartment (*K*_1_ and *k*_2_) (C), and 2T6P rate constants between first and second compartments (*k*_3_ and *k*_4_) (D).

When the pre- and posttherapy scans of the NSCLC patient were compared, SUVR, *K*_Logan_, and *V*_T_ showed significant increases (*P* = 0.016) in all 7 analyzed subregions of the LLL tumor after therapy, in addition to significantly increased *k*_3_ (*P* = 0.047) and significantly decreased *k*_4_ (*P* = 0.016) (Fig. 7), with no significant change in *K*_1_ and *k*_2_. The changes of SUVR, *K*_Logan_, and *V*_T_ of the mediastinal LNs were different between the 2 enlarged LNs and the 2 nonenlarged LNs, with decreasing and increasing trends after therapy, respectively (Supplemental Fig. 9). Lastly, the analyzed axillary and pelvic LNs of the NSCLC patient also showed a significant increasing trend (*P* < 0.0001) in *k*_3_ after therapy, as well as a significant decreasing trend (*P* = 0.045) in SUVR; however, no significant changes in *K*_Logan_ and *V*_T_ were observed (Supplemental Fig. 10).

Additional data on the results of the AIC model selection, normalized sensitivity plots, practical identifiability analysis, SUVR curves, Logan plots, and scatterplots of all dataset pairs investigated with the Spearman rank correlations are available elsewhere (11).

## DISCUSSION

Total-body late–time-point biodistributions were in good agreement with those from previous static [^18^F]F-AraG scans in healthy volunteers (8). However, the high sensitivity of total-body PET resulted in visualization of a notably larger number of LNs in all subjects of this study, despite the 40% reduction in injected radiation dose compared with that in previous in-human studies. This is of particular interest for evaluating the systemic immune response to therapeutic interventions in cancer patients, because of the critical role of tumor-draining LNs, but also in other disease conditions that stimulate the immune system.

Tumor *immune contexture* has been previously proposed to describe the nature, density, immune functional orientation, and distribution of immune cells within the tumor and has been associated with long-term survival and treatment response prediction (18*,*^19^). The tumor immune contexture has been increasingly used for classification of tumors into major categories of hot, altered, and cold tumors (18). In the context of using [^18^F]F-AraG imaging as a biomarker for immunotherapy response evaluation, 3 major factors are considered to play key roles in this study: first, the initial [^18^F]F-AraG scan serving as a biomarker for quantification of the baseline antitumor immune response and tumor immune contexture classification; second, changes in tumor uptake in posttherapy [^18^F]F-AraG scans serving as biomarkers of therapy-induced alterations in the tumor microenvironment; and third, systemic quantification particularly in tumor-draining LNs, as important sites of antigen presentation and T-cell activation. Although [^18^F]F-AraG SUV images could serve for tumor classification as previously shown in preclinical studies (10), parameters obtained by kinetic modeling, or their surrogates, are expected to provide higher quantification accuracy especially when different scans are compared, primarily because of differences in time-varying blood input function (particularly, variabilities in the blood clearance) and its subsequent effects on tissue SUVs. Analyzing 7 subregions of a NSCLC tumor in this study has shown promising results in evaluating the therapy-induced changes in the tumor microenvironment. Despite the variability observed in tumor SUV_mean_ changes after therapy, the SUVR, *K*_Logan_, and *V*_T_ showed increasing trends in all analyzed subregions of the tumor. Furthermore, the significant increasing trend in *k*_3_ and the decreasing trend in *k*_4_ observed in posttherapy scans of the tumor are in good agreement with the expected immunotherapy-induced increase in T-cell activation, leading to higher dGK and lower SAMHD1 expression in the activated T cells infiltrating the tumor (7*,*^8^). These findings are also consistent with the slight tumor-size reduction evidenced on CT scans, suggesting an initial effectiveness of the therapy; however, they require future investigation in a larger cohort of cancer patients.

The differences observed in tumor uptake patterns between [^18^F]FDG and [^18^F]F-AraG are consistent with previous preclinical studies (7) and can be attributed to fundamental differences in the source of uptake in [^18^F]FDG and [^18^F]F-AraG scans. The large contribution of tumor cell metabolism to the [^18^F]FDG signal prevents accurate quantification of the immune infiltration within the tumor microenvironment, whereas a major fraction of [^18^F]F-AraG uptake in NSCLC tumors is expected to originate from the immune cells in the tumor (7). However, the 3.5-mo interval between the [^18^F]FDG and the [^18^F]F-AraG scans in this study may have also contributed to the tumor progression.

The substantial increase of SUVR, *K*_Logan_, and *V*_T_ observed in the 2 nonenlarged mediastinal LNs and the decreasing trend observed in the 2 enlarged mediastinal LNs may be explained by differences in therapy response of uninvolved and involved regional LNs (20); however, it requires further investigation in a larger cohort of patients, especially considering the slight size reduction of the 2 enlarged nodes on posttherapy CT. Given that the identifiability analysis did not show substantial increased bias or variability in the mediastinal LNs compared with other organs, the current findings suggest that quantitative response assessment in tumor-draining LNs might require multiple longitudinal scans after therapy to consider the effects from T-cell trafficking (2). Interestingly, the axillary and pelvic LNs of the patient showed a significant increasing trend in *k*_3_, which is consistent with the expected global effect of anti–PD-1 immunotherapy on peripheral T cells, distant from the primary tumor.

Promising kinetic modeling results obtained in all investigated organs of interest suggest that [^18^F]F-AraG imaging can be used as a quantitative biomarker for a wide range of applications, such as graft-versus-host disease, arthritis, multiple sclerosis, and viral infections. This is further supported by the incidental findings of the study, showing increased uptake in potential sites of immune infiltration, such as arthritic joints, meningioma, the colon wall, and the underarm skin. The AIC analysis favored the reversible 2-tissue compartmental model across various organs of interest with the tested high-temporal-resolution framing protocol. The kinetic modeling was validated through the strong correlations observed between (SUVR, $V_{T\left(v_{b}\right)})$ and (*K*_Logan_, $V_{T\left(v_{b}\right)})$ in all organs of interest. Furthermore, the proximity of $V_{T\left(v_{b}\right)}$ estimates to extrapolated SUVR curves at 10 h after injection is suggestive of $V_{T\left(v_{b}\right)}$ estimation accuracy, and the remaining differences observed between the $V_{T\left(v_{b}\right)}$ and the converged SUVR curves at equilibrium are possibly from biexponential fitting errors on whole-blood time–activity curves, which requires future validation using delayed imaging. The extrapolated SUVR curves suggest that equilibrium may be reached 4 h after injection in the analyzed organs of interest with substantial differences in the organ equilibrium times, as expected.

The correlations of *K*_Logan_ and SUVR with *V*_T_ were generally strong, suggesting that these measures could serve as surrogates for *V*_T_ estimation without kinetic modeling. However, their use may require careful attention in organs with high blood-volume fraction, such as myocardium. SUV_mean_ from 60 to 90 min after injection showed moderate to strong correlations with *V*_T_ in a few organs only, including the tumor subregions, which indicates their potentially limited utility for quantification of tracer uptake. However, it has to be noted that kinetic analysis of all tumor subregions of this study suggested low blood-volume fraction in all of the subregions, and consequently, weaker correlations of *K*_Logan_ and SUVR could be expected possibly in other tumor types with higher blood-volume fraction. The present study recommends the use of SUVR over SUV in static scans in which kinetic modeling or graphical analysis methods may not be feasible. The negative correlation observed between lung SUV_mean_ and *V*_T_ requires further investigation with a larger sample size but also highlights the potential misleading nature of SUVs in certain cases. Future work on evaluating the correlations of SUV and SUVR in different organs of interest with *V*_T_ as a function of uptake time is required for optimizing the acquisition time on clinical scanners with a short axial field of view.

The differences observed between the 2 *K_i_* estimates and the variability of the correlation results in different organs suggest that *k*_4_ effects may not be negligible in many organs of interest. In tissues where activated T cells are expected to be dominating factors in the tracer kinetics, such as the tumor, LNs, or the investigated joints of the 2 control subjects in this study, the difference between the 2 *K_i_* estimates is smaller, and stronger correlations with SUVR are observed. However, even in such cases, SUVR still correlates more strongly with *V*_T_ and $V_{T\left(v_{b}\right)}$, and using a reversible model is recommended.

The practical identifiability analysis suggests that *V*_T_ can be estimated reliably in all organs of interest with low bias and variability. The individual rate-constant estimates also showed low biases but had larger variabilities than *V*_T_, particularly in healthy organs, which may require larger sample sizes when used for hypothesis testing. The improved results obtained by fixing the time-delay values suggest that variabilities in time-delay estimation may largely affect the input-function properties, leading to increased variabilities in microparameter estimation. Therefore, future studies should investigate the use of improved time-delay estimation methods. Although a continuous increasing trend in *k*_4_ sensitivity was observed during the 90-min scans in all organs of interest, the identifiability analysis results obtained with 60-min time–activity curves suggest sufficient sensitivity for *k*_4_ in the first 60 min for reducing the dynamic scan duration in future studies. Furthermore, the similarity of *k*_4_ estimates obtained with 60-min and 90-min dynamic data suggests negligible effects from the time-variant plasma fraction and parent fraction on *k*_4_ estimation.

This pilot study had some limitations. The small sample size and the inclusion of a single NSCLC patient warrant further investigations in larger cohorts. Particularly, the results of therapy response evaluation, which relied on data from a single tumor in a single patient, are not generalizable. This necessitates cautious interpretation of the current findings and requires validation with various tumor types in a larger cohort of patients. The use of graphical analysis with the Logan plots requires further investigation, particularly to optimize the t* in different organs of interest. Furthermore, future work on validation of the dynamic imaging protocol and the proposed kinetic model for [^18^F]F-AraG should include delayed imaging beyond 90 min after injection. Future studies should also investigate the effect of tracer uptake by immune cells present in whole blood on the kinetic modeling by longitudinal blood sampling and should quantify the tracer metabolites in blood and their effects on kinetic parameters. Lastly, the currently used compartmental modeling may not be optimal for some organs and tissues, such as the liver, RV myocardium, and certain types of lung tumors, and the models can be further optimized by incorporating additional factors, such as dual input function, spillover, dispersion, and air-fraction correction.

## CONCLUSION

This study demonstrates the potential of [^18^F]F-AraG dynamic imaging as a noninvasive early biomarker for evaluating the immune response to therapeutic interventions in cancer patients and underscores the importance of quantification methods, such as kinetic modeling or graphical methods, for accurate assessment of immune contexture and therapeutic response prediction. Increasing trends were observed in SUVR, *K*_Logan_, and *V*_T_ in all analyzed subregions of the tumor after therapy, whereas SUV_mean_ changes varied in different subregions of the tumor. The correlations between *K*_Logan_ and SUVR with *V*_T_ show promise as surrogates for *V*_T_ estimation, especially in organs with low blood-volume fraction. In addition, the study suggests that the dynamic [^18^F]F-AraG PET scans could potentially be shortened to 60 min, while maintaining quantification accuracy for all organs of interest. Lastly, the incidental findings of the study, consistent with the medical history of the subjects, further highlight potentially wider applications of the tracer in investigating the role of T cells in the immunopathogenesis of diseases.

## DISCLOSURE

This work was supported by CellSight Technologies and National Institutes of Health grants R01CA206187 and R35CA197608. Jelena Levi is employed by CellSight Technologies. CellSight Technologies is commercializing [^18^F]F-AraG. No other potential conflict of interest relevant to this article was reported.

## Notes

KEY POINTS **QUESTION:** Do kinetic parameters, estimated from total-body dynamic imaging of [^18^F]F-AraG, provide expanded information on sites of T-cell activation? **PERTINENT FINDINGS:** This pilot study has demonstrated the feasibility of [^18^F]F-AraG kinetic modeling and high identifiability of kinetic parameters in different tissue types. Furthermore, in a lung tumor, [^18^F]F-AraG kinetic parameters suggested T-cell activation (with significant increases in *V*_T_ and *k*_3_ and a significant decrease in *k*_4_), consistent with a slight size reduction of the tumor after therapy evidenced on CT, whereas SUVs did not show any significant changes after therapy. **IMPLICATIONS FOR PATIENT CARE:** This study introduces [^18^F]F-AraG dynamic imaging and kinetic modeling as a noninvasive quantitative biomarker for evaluating the immune response to therapeutic interventions in cancer patients.
